# Behçet’s Disease: Do Natural Killer Cells Play a Significant Role?

**DOI:** 10.3389/fimmu.2015.00134

**Published:** 2015-03-24

**Authors:** Harry Petrushkin, Md. Samiul Hasan, Miles R. Stanford, Farida Fortune, Graham R. Wallace

**Affiliations:** ^1^Clinical and Diagnostic Oral Sciences, Queen Mary University of London, London, UK; ^2^Academic Unit of Ophthalmology, St Thomas’s Hospital, London, UK; ^3^Centre for Translational Inflammation Research, University of Birmingham, Birmingham, UK

**Keywords:** Behçet’s syndrome, Behçet’s disease, Behçet’s, NK cells, KIR3DL1, KIR, HLA-B*51, HLA-B antigens

## Abstract

Behçet’s disease (BD) is a complex inflammatory disease, of unknown etiology. While disease pathogenesis remains unclear, a strong relationship between BD and HLA-B*51 has been established over the last 30 years. A number of theories exist regarding the cause of BD; however, few are able to account for the increased rates of HLA-B*51 positive individuals, particularly around the Mediterranean basin and Middle-East where the prevalence is highest. This review outlines current immunogenetic data on BD and the immunoregulatory role natural killer cells may play. It also describes the interaction of the killer immunoglobulin-like receptor – KIR3DL1 with its ligand Bw4, which is found on HLA-B51. Finally, CD94/NKG2D, MICA, and ERAP are outlined with regard to their potential roles in BD.

## Behçet’s Disease

Behçet’s disease (BD) is a complex systemic inflammatory disorder, consisting of oral aphthous ulcers, genital ulcers, papulopustular and erythema nodosum-like skin lesions, uveitis, retinal vasculitis, thrombophlebitis, arterial aneurysms, and arthritis. The disease is found primarily along the ancient Silk Route from the Mediterranean Basin across Asia to Japan (1, 2). The prevalence of BD in UK is around 0.64/100,000, whereas in Istanbul 370/100,000 individuals are affected by this disease (3).

## Natural Killer Cell Biology

Natural killer (NK) cells are lymphocytes of the innate immune system. They are involved in the early stages of defense against foreign and self-cells undergoing stress. NK cells comprise 10–15% circulating lymphocytes in humans, falling mostly into two distinct populations. About 90% are CD56^dim^CD16^+ve^ cells, capable of killing and antibody dependent cytotoxicity. These cells produce relatively little IFNγ (4, 5). NK cells identify their targets through a set of activatory or inhibitory receptors, which recognize non-self targets as well as self-proteins that are upregulated as a result of stress. They can also kill cells that down regulate expression of self-MHC molecules during times of infection or transformation (6, 7).

The other 10% are CD56^bright^CD16^−ve^ and are weakly cytotoxic. These cells do not proliferate vigorously in response to IL-2, but produce substantial amounts of IFNγ, granulocyte–macrophage colony-stimulating factor (GM-CSF), chemokines such as chemokine ligand 3 (CCL3) and CCL4 (8). It has been suggested that CD56^bright^CD16^−ve^ cells utilize their cytokine production to play a regulatory role in immune responses while CD56^dim^CD16^+ve^ cells are terminally differentiated cytotoxic effector cells (9).

Natural killer cells express a number of activatory and inhibitory receptors. Killer immunoglobulin-like receptors (KIR) and NKG2D are discussed in detail below with respect to their role in BD. Natural cytotoxicity receptors (NCR) are activatory receptors found on NK cells. There are three known receptors; NKp46, NKp30, and NKp44. NKp46 and NKp30 are constitutively expressed, whereas NKp44 is expressed during NK cell activation by IL-2 (10, 11). NCR have been investigated in the context of tumor biology and cytokine production; however, they do not interact directly with class-I ligands and there is limited evidence for their role in autoimmune/autoinflammatory disease.

IL-15 and IL-15Rα are essential for normal development of naive CD8^+ve^ T cells, intestinal intraepithelial lymphocytes, and NK cells. IL-15 shares structural similarity with IL-2 and both cytokines signal through CD122 to increase proliferation of NK cells (12).

Elevated IL-15 levels have been reported in serum, cerebrospinal fluid, and aqueous humor from patients with BD (13–15). Serum IL-15 levels have been found to correlate with erythrocyte sedimentation rate but not C-reactive protein levels and IL-15Rα expression has been found to be significantly reduced in leukocytes from patients compared to controls (12). While these findings suggest a possible role for IL-15 in BD pathogenesis, the mechanism remains unclear. It would be useful to establish whether there is a link between BD clinical activity and serum/organ specific IL-15/IL-15Rα levels. The effect of altered levels of IL-15 and the IL-15R complex is known to impact NK cell proliferation thus suggesting a possible pathway for NK cell control in the disease.

## NK Cell Licensing

Natural killer cells are “licensed” to recognize and kill cells that do not express self-MHC. In 1986, Karre et al. described this phenomenon as the “missing self” hypothesis (16). This states that NK cells are activated to detect and kill cells that are thought to be non-self, i.e., infected cells or those undergoing neoplastic changes. Over the last 30 years, a number of authors have demonstrated that NK cells from MHC-deficient mice and humans do not effectively kill target cells (17, 18).

Natural killer cell receptors such as KIR have loci that segregate independently of MHC loci. Thus, in any one individual, some NK cells do not have a corresponding inhibitory HLA ligand, i.e., KIR3DL1^+ve^ cells in a Bw6 homozygote. It is unclear how such cells could be prevented from killing autologous cells. Yokoyama et al. proposed that KIR on NK cells must recognize their cognate HLA ligand in order to acquire functional competence through licensing (19). An alternate “disarming” hypothesis proposes that NK cells that fail to recognize MHC class-I via inhibitory KIR become anergic (20). Hence, paradoxically, an inhibitory receptor interaction appears to be required for an NK cell to acquire function.

In the context of infection, unlicensed NK cells may become activated in response to cytokines from infected cells. This may permit a broader repertoire of NK cells to contribute to the response to pathogens without the risk of autoimmunity (21). Moreover, the licensing concept embraces gene dosage, i.e., KIR3DL1^+ve^ cells in donors who are homozygous for Bw4 display increased responsiveness to tumor stimulation compared to heterozygotes and those who lack the ligand. In contrast, NK cells lacking KIR3DL1 show no difference in activity (22).

## Genetic Studies in Behçet’s Disease

**Figure 1:**
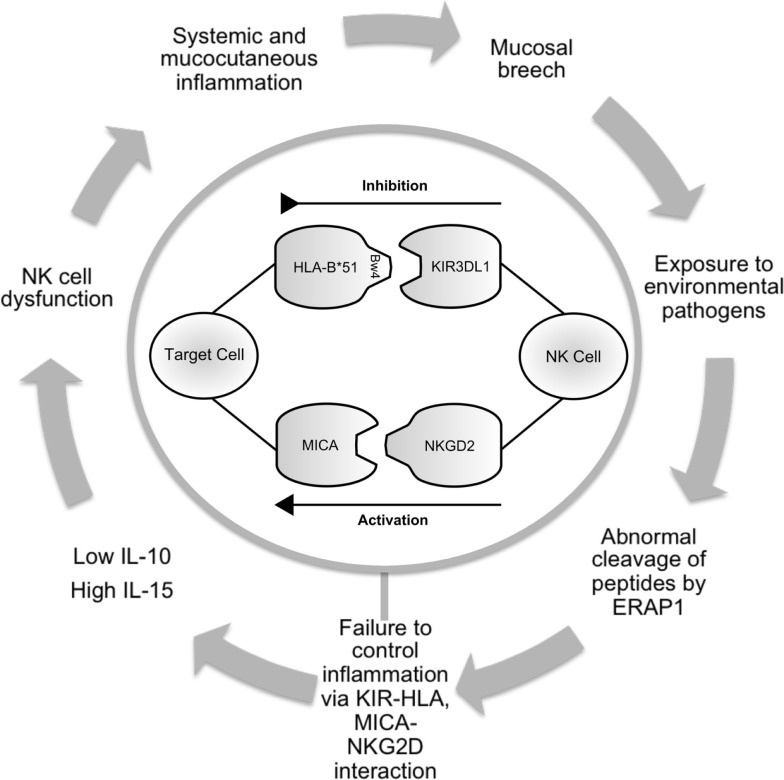
**Schematic illustrating the potential role of NK cells in Behçet’s disease**. Infection or inflammation at mucocutatneous surfaces may persist due to dysfunction of the innate immune response in predisposed individuals. ERAP1 may have a role in processing peptides that are presented by HLA-class-I molecules. KIR3DL1 inhibits cell-mediated cytotoxicity via interaction with HLA-B*51. This effect is balanced by activatory interactions via NKG2D and MICA. Production of cytokines including IL-10 and IL-15 contribute to abnormal NK cell function leading to prolonged inflammation and further episodes. NK cell, natural killer cell; ERAP, endoplasmic reticulum aminopeptidase 1; HLA, human leukocyte antigen; KIR, killer immunoglobulin-like receptor; MICA, MHC class-I polypeptide-related sequence A; NKG2D, natural killer group 2; receptor D; IL, interleukin.

Evidence for a genetic association in BD first came about in 1982, when Ohno et al. published their results suggesting that HLA-B*51 was associated with BD in Japanese patients (23). This association has become recognized as the most frequently observed genetic factor in BD and is present in between 13 and 80% of patients (24). HLA-B51 presents antigen to CD8^+ve^ cytotoxic T cells, but is also known to interact with the KIR3DL1 via its Bw4 epitope (25) (Figure 1). KIR interacts with position 7 and 8 of the HLA-bound peptide and with positions 77–83 of HLA molecules that have the Bw4 epitope (26, 27). Bw4 is present on a third of all HLA-B alleles and is defined by leucine at position 82 and arginine at position 83. It is associated with strong inhibition of NK cell activity via KIR3DL1 interaction. The Bw6 epitope (Ser 77, Asn 80, Leu 81, Arg 82, and Gly 83) does not interact with KIR3DL1 (28). There is considerable variation within the KIR haplotype. *KIR* genes differ in content and copy number and not all alleles are present in every individual.

The Bw4 epitope can be found on other *HLA* alleles associated with BD, such as HLA-B*2702 (29) as well as those thought to be protective, such as HLA-B*58 or HLA-A*0301 (30). The association with HLA-B*51 may be due to peptide binding with low affinity leading to reduced tolerance and/or inefficient NK cell licensing (31).

Four genome-wide association studies (GWAS) investigating genetic associations in BD have been carried out to date and all have confirmed association with the MHC region (32–35). In the last 3 years, data from these studies have been subjected to imputational analysis (a process using known haploytpes to allow testing of untyped genetic variants for association with a trait of interest) and a number of genes were found to achieve significance including: C–C motif chemokine receptor 1 (*CCR1*), *STAT4*, killer cell lectin-like receptor subfamily C (*KLRC4*), and endoplasmic reticulum amino peptidase 1 (*ERAP1*) (35). All of these genes are involved in cytotoxicity in some capacity. Of note, *KLCRC4* is located on a haplotype block, which also codes for other NK cell receptors, such as NKG2D (36). A functional role for this gene has yet to be established, but there is evidence for reduced cytotoxicity in NK cells from patients carrying this haplotype (37) (Table 1).

**Table 1:** **Genes implicated in the pathogenesis of Behçet’s disease and their potential mechanisms of action**.

Genes implicated in the pathogenesis of BD	Mechanism of interaction with NK cells	Overall effect on NK cells
HLA-B*51 (HLA-B*5101) (38)	Interaction with KIR3DL1 via the Bw4 epitope (39)	Inhibition
MICA (MICA*009) (40–43)	Interaction with NKG2D (44)	Activation
IL-23R/IL-12Rβ2 (33, 34)	Down regulated gene expression of IL-12Rβ2 (45)	Rendered hyporeactive to IL-12 with deficient IFNγ production
IL-10 (33, 34)	Unknown	Possible effect on increasing pro-inflammatory cytokine expression and increasing cytotoxicity
KLRC4 (35)	Unknown	Possible effect on reducing peripheral blood cytotoxicity in NK cells (37)
CCR1 (35)		
ERAP1 (35)	Trims proteasome-derived peptides before loading into HLA-class-I binding groove	Alters recognition of HLA-class-I by KIR

In 2013, Hughes et al. closely examined the HLA region using deep sequencing, and identified rs116799036, a SNP in the HLA-B*51 promoter region, 24 kb upstream from HLA-B*51 and 18 kb upstream of *MICA*, which was found to achieve genome-wide significance even when controlled for the effects of HLA-B*51. Furthermore, when controlling for the effects of rs116799036, the association with HLA-B*51 was abrogated, suggesting that the association between HLA-B*51 and BD may not be causal (38). However, this finding was not replicated in a larger cohort, which confirmed HLA-B*51 as the strongest association (46). In addition, this study found that HLA-A*03, HLA-B*15, HLA-B*27, HLA-B*49, HLA-B*57, and HLA-A*26 each contributed independently to the risk of developing BD. This data support HLA-B*51 as the strongest association in BD.

## Functional Activity of NK Cells in Behçet’s Disease

In order to investigate the functional activity of NK cells in BD, Yamaguchi et al. assessed 47 patients with BD (10 with active disease). CD69^+ve^ NK cells were significantly increased in active BD, but their cytotoxic function was similar to inactive and control subjects. IL-12Rβ2 mRNA levels were decreased in NK cells from patients with active BD, compared to inactive patients or healthy controls. Furthermore, NK cells from inactive patients suppressed IFNγ production by CD4^+ve^ T cells from patients with active disease (45). These findings suggest that while NK cells proliferate in active BD, they may be rendered hyporesponsive due to an abnormality in IL-12 signaling. Hamzaoui et al. also described hyporesponsive NK cells in bronchoalveolar lavage (BAL) samples from patients with pulmonary BD. The authors did not examine the effects of IL-12, but instead focused on IL-2/IL-15Rβ (CD122), which was found to have reduced surface expression. CD122 is shared by the IL-15 receptor, which is partly responsible for maintenance and cytotoxicity of NK cells (47).

Treusch et al. evaluated the effect of IFNα-2a on the NK cell repertoire in BD. IFNα-2a is often used as a second or third line therapeutic agent in BD, particularly in the presence of ocular disease. The investigators found a significant decrease in circulating NK cells in active BD patients after treatment compared to controls. This reduction may be a direct effect of IFNα-2a or secondary induction of remission of BD. IFNα-2a has been postulated to work directly on NK cells by inducing apoptosis and indirectly by reducing IL-18, IL-12, and/or IL-21 (48).

## NK Cell Receptors

### Killer immunoglobulin-like receptors

In humans, receptors belong to either the immunoglobulin-like superfamily (IgSF) or the C-type lectin-like receptor (CTLR) superfamily. KIR are part of the IgSF and have specific ligands in the HLA-class-I family (49).

Inhibitory KIR (i.e., KIR2DL and KIR3DL), contain immunoreceptor tyrosine-based inhibition motifs (ITIMs) in their cytoplasmic domains. Activating KIR molecules (i.e., KIR2DS and KIR3DS) lack ITIMs and have a charged residue in their transmembrane domains, which likely pair with the DAP12 signaling adapter. KIR2DL4 is unusual as it consists of a long intracellular region but only one ITIM and a positively charged amino acid in the transmembrane region. Unlike other clonally distributed KIR, KIR2DL4 is transcribed by all NK cells and acts as an activating receptor on recognition of HLA-G (50, 51). Inhibitory KIR consistently binds with a higher affinity than their activating counterparts (52).

Saruhan-Direskeneli et al. found no association of KIR3DL1 expression on NK or T cells in patients with BD, a finding not altered by the presence of Bw4 motif in patients (53). Similar results were found by Middleton et al. after analyzing 14 *KIR* genes in a cohort of Turkish patients and ethnically matched controls using a sequence-specific oligonucleotide probe (SSOP) method. They reported an increased frequency of KIR3DL1 and its ligand Bw4 (*p* = 0.0003) and a corresponding decreased frequency of KIR3DL1 without Bw4 (*p* = 0.00004) in patients compared to controls. However, this difference was abrogated when the presence of HLA-B*51 was controlled for (*p* = 0.7075) (54).

The frequency of 16 *KIR* genes (*2DL1*, *2DL2*, *2DL3*, *2DL4*, *2DL5*, *3DL1*, *3DL2*, *3DL3*, *2DS1*, *2DS2*, *2DS3*, *2DS4*, *2DS5*, *3DS1*, *2DP1*, and *3DP1*) was assessed in Korean patients with either HLA-B27-associated ankylosing spondylitis and uveitis or BD related uveitis. In the patients with ankylosing spondylitis, the frequency of *KIR3DL1* was significantly lower than healthy controls (*p* = 0.043), while there was no difference in patients with BD (55).

These studies support a role for KIR3DL1 and Bw4 in BD; however, there is currently insufficient evidence to suggest that this is via KIR3DL1–HLA-B51 interaction. The *KIR* cluster is highly polymorphic, second only to the *HLA* in complexity and is an unsuitable candidate for GWAS analysis as SNP probes targeting the region are unable to bind due to allelic and copy number variation within the gene. Other methods including targeted sequencing and functional analysis of different alleles must be explored further.

### CD94/NKG2

The CD94/NKG2 heterodimer is a member of the CTLR family and recognizes HLA-E (56). It is thought that CD94/NKG2 work in conjunction with KIR by responding to changes in HLA expression and can also transduce both activating and inhibitory signals (57). The ligand for CD94/NKG2A is the non-classical HLA-E. Peptides bound to HLA-E function as modulators of NK cell activity. The NKG2D homodimer, is expressed at various levels on NK cells and recognizes MICA and UL16-binding protein (ULBP) leading to NK cell activation through DAP10 signaling.

Saruhan-Direskeneli et al. found CD94 expression was increased on CD56^+^CD16^+ve^ and CD56^+^CD3^+ve^ NK cells in patients with BD (53). It has been subsequently demonstrated that both *CD94* (c.-134*T) and *NKG2A* (c.-4258*C, c.338-90*G) polymorphisms are associated with reduced risk of BD, an effect enhanced when combined with expression of HLA-E*0101. Individuals without this genotype had nearly a fivefold increased risk of developing BD. Furthermore, the NKG2C c.305*T polymorphism resulted in an increased rate of both ocular disease and arthritis (*p* = 0.0001), whereas the CD94 c.-134*A polymorphism was associated with oral, skin, genital, and gastrointestinal manifestations (58). Of note, HLA-E binds peptides derived from HLA-class-1 molecules and are also recognized by NK cells via CD94/NKG2A, resulting in an inhibition of cell lysis, thus providing another mechanism for NK cell regulation (59).

While frequently overlooked in the field of BD, HLA-E, and HLA-G have important roles in regulating inflammation. HLA-E is an activator of NK cells via its interaction with CD94/NKG2A and HLA-G inhibits the cytolytic activities of NK cells via its interaction with KIR2DL4 and LIR-1/LIR-2. A study in Korean patients with BD indicated that the variants; HLA-E*0101 and HLA-G*010101 (*p* = 0.0002, OR 0.7, *p* = 0.002, OR 0.7, respectively) were associated with a reduced risk of developing the disease, whereas HLA-E*010302, HLA-G*010102G*0105N alleles, and *3741_3754ins14bp* were all associated with an increased risk of BD (*p* < 0.0001, OR 1.6; *p* = 0.002, OR 1.8; *p* = 0.024, OR = 2.0; and *p* = 0.003, OR 1.4, respectively). This data indicates that target cells expressing HLA-E*0101 and HLA-G*010101 are protected against NK and CD8^+ve^ cell-mediated cytotoxicity. Conversely polymorphisms in HLA-E and HLA-G may lead to an imbalance of lymphocyte functions and to the development of BD (60).

## MICA in Behcet’s Disease

MICA is a stress-induced antigen and is recognized by the lymphoid stress surveillance system. It is a ligand for NKG2D, and involved in NK cell activation (Figure 1). While it is generally agreed that the association between MICA and BD is largely due to linkage disequilibrium (LD) with HLA-B*51 (61–63), MICA may have an independent functional role in disease pathogenesis. In Korean patients, MICA*A6 homozygosity was associated with BD in both HLA-B*51^+ve^ and HLA-B*51^−ve^ patients. The authors also assessed the susceptibility risk of MICA*A6 homozygosity for BD in HLA-B*51^−ve^ patients and found an independent association (*p* < 0.001, RR-22.27) (64).

The affinity of MICA as a ligand for NKG2D is not uniform. Low affinity MICA have a valine at codon 129 and include MICA*019 and MICA*009; these receptors have a 10- to 50-fold weaker affinity for NKG2D compared to those with methionine in position 129 (65). Moreover, an individual’s response to different MICA alleles is “tuned” and maintained over time (44).

Interestingly Munoz-Saa et al. commented that none of their 42 BD patients expressed a high affinity phenotype for NKG2D compared to 14% of controls, whereas other autoimmune diseases such as anterior uveitis, ankylosing spondylitis, and psoriatic arthritis are all associated with high affinity alleles (42, 66–68).

The association of MICA and MHC class-I haplotypes with BD, suggests a major role in the disease for cells regulated by these molecules including NK cells and subsets of T cells (40, 69).

## Endoplasmic Reticulum Aminopeptidase

As discussed above, a polymorphism at the ERAP1 locus was associated with BD by imputation of GWAS data (35). The most documented function of ERAP1 and ERAP2 is to trim peptides that bind to MHC class-I molecules (Figure 1). Different ERAP1 variants can produce a different peptide pool, which in turn can affect folding and expression of MHC class-I on the cell surface and CD8 T-cell cytotoxicity. Furthermore, KIR and CD94/NKG2A function are affected by the peptide bound to MHC class-I (70). At present, there is no published data reporting the functional effects of endoplasmic reticulum aminopeptidase (ERAP) polymorphisms in BD. Future work addressing this area is warranted.

## Conclusion

Several potential mechanisms explaining the pathogenesis of BD involve NK cells:

1. Defects in the NK cell repertoire may permit persistent viral infections, resulting in a chronic inflammatory response leading to BD.
2. NK cells lacking appropriate inhibitory KIR (such as KIR3DL1) may fail to recognize self-MHC and cause autologous tissue damage (71).

The role of NK cells in the pathogenesis of BD has been greatly advanced by genetic studies and the association of MICA and ERAP support involvement of the innate immune system.

However, major challenges remain, such as the lack of association between inhibitory KIR receptors and HLA-B51. I. In the last decade, over 50 new *KIR3DL1* allotypes have been described, only a small proportion of which are present in different world populations (28, 72). It would be useful to establish the geographic spread of *KIR3DL1* and correlate it to previously published work on HLA-B*51 (2).

It is possible that future work investigating *KIR3DL1* polymorphisms will uncover allotypes previously untested for. Such studies, carried out in well-defined and characterized cohorts with adequate numbers to confirm the basic findings, to date, will not only strengthen the hypotheses outlined in this review but also create a body of evidence that may lead to potential new therapies.

## Conflict of Interest Statement

The authors declare that the research was conducted in the absence of any commercial or financial relationships that could be construed as a potential conflict of interest.
